# Anatomical and functional outcomes of pneumatic vitreolysis for treatment of vitreomacular traction with and without macular holes

**DOI:** 10.1007/s00417-022-05568-y

**Published:** 2022-02-05

**Authors:** Carmen Baumann, Francesco Sabatino, Yalin Zheng, Navid Johannigmann-Malek, Mathias Maier, Stephen B. Kaye, Niall Patton

**Affiliations:** 1 Ophthalmology Department, Hospital rechts der Isar, Technical University of Munich (TUM), Ismaninger Str. 22, 81675 Munich, Germany; 2 Manchester Royal Eye Hospital, Oxford Road, Manchester, M13 9WL UK; 3 University of Liverpool, William Henry Duncan Building, 6 West Derby Street, Liverpool, L7 8TX UK

**Keywords:** Pneumatic vitreolysis, Vitreomacular traction, Macular hole

## Abstract

**Purpose:**

To evaluate the outcome of pneumatic vitreolysis (PVL) for vitreomacular traction (VMT) with or without full thickness macular hole (MH) < 400 µm.

**Methods:**

Forty-seven eyes of 47 patients were included who had undergone PVL for VMT with or without MH. Main outcome measures were release of VMT, MH closure, best-corrected visual acuity (BCVA) and adverse events.

**Results:**

Thirty-three patients had isolated VMT and 14 patients VMT with a MH. Four weeks after PVL, the overall VMT release rate was 35/47 (74.5%): 25/37 (67.6%) in phakic and 10/10 (100%) in pseudophakic eyes (*p* = 0.03). Four of 14 MH (28.6%) were closed. Twenty-two of 47 (46.8%) eyes required a subsequent PPV: 12/33 (36.4%) in the VMT only group and 10/14 (71.4%) in the VMT with MH group. Mean BCVA improved from 0.48 (± 0.24) to 0.34 (± 0.23) logMAR at 6 months in patients with VMT alone (*p* < 0.001), and from 0.57 (± 0.27) to 0.41 (± 0.28) logMAR in patients with VMT and MH (*p* = 0.008). Adverse events included new formation of a large MH in 4/33 (12.1%) eyes, failure of MH closure in 10/14 (71.4%) eyes, progression of mean minimum linear diameter (MLD) MH size from baseline 139 (± 67) to 396 (± 130) µm (*p* < 0.001) and development of a retinal detachment in 4/47 (8.5%) eyes.

**Conclusion:**

While PVL leads to a high VMT release rate particularly in pseudophakic eyes, it is associated with a relatively high incidence of MH formation, MH size progression and retinal detachment.


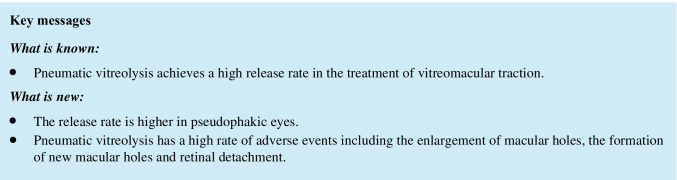


## Introduction

Vitreomacular traction (VMT) is the result of an abnormal posterior vitreous detachment (PVD) characterised by persistent adherence of the vitreous to the central macula in the presence of partial vitreous separation from the adjacent retina. The resultant macular traction may lead to a decrease in central vision and metamorphopsia, potentially culminating in macular hole (MH) formation [1]. Only around 10–32% of cases resolve spontaneously [1–3], the remainder often requiring treatment, particularly if a MH has formed.

The standard treatment to release VMT has been pars plana vitrectomy (PPV). Pharmacological treatment in the form of intraocular injection of ocriplasmin has been found to promote liquefaction and separation of the vitreous from the retina due to its proteolytic activity on the vitreoretinal interface [4, 5] and was approved by the Food and Drug Administration in 2012 for VMT with and without MH ≤ 400 µm. However, the early enthusiasm for ocriplasmin was dampened by the initially reported low success rate in PVD induction of 26.5% [5, 6] though in particular subgroups of patients, the rate of PVD induction can be significantly higher. In addition, there have been reports of multiple though mostly transient side effects including vision loss, macular edema, dyschromatopsia accompanied by changes on ERG and on the photoreceptor layer on optical coherence tomography (OCT). There have also been reports of retinal breaks with consequent rhegmatogenous retinal detachment (RD). [6–10].

An alternative treatment termed pneumatic vitreolysis (PVL), first described by Chan et al. [11] in 1995, aimed to achieve mechanical induction of PVD by intravitreal injection of an expansile gas bubble. However, despite reported high VMT release rates, easy accessibility and low cost of this procedure, only a few reports on PVL exist, predominantly in eyes without MH and comprising heterogeneous patient cohorts. [11–19].

The aim of this study was therefore to analyse the efficacy and risk profile of PVL in the treatment of both VMT alone and VMT associated with macular hole (MH), in the absence of any other retinal co-pathology.

## Materials and methods

This was a retrospective, non-randomised interventional case series of consecutive patients who underwent pneumatic vitreolysis (PVL) for the treatment of symptomatic isolated VMT or VMT with macular hole (MH) ≤ 400 µm at the Ophthalmology Department, Hospital rechts der Isar, Technical University of Munich, Germany, and the Manchester Royal Eye Hospital, UK, over a 4-year period between 07/2016 and 06/2020. The diagnosis of VMT with or without MH was confirmed by fundoscopy and OCT (Spectralis HRA + OCT, Heidelberg Engineering, Heidelberg, Germany or 3D-OCT 2000, Topcon Medical Systems, N, USA).

Inclusion criteria were presence of VMT ≤ 1500-µm adhesion length with or without a MH ≤ 400 µm. Exclusion criteria were previously treated VMT, persistent and secondary MH, MH > 400 µm, myopia > 8.00 diopters, the presence of an epiretinal membrane, significant macular degeneration, diabetic retinopathy, preexisting peripheral retinal pathology predisposing to retinal breaks or a history of retinal tears or detachment in either eye, or any other relevant retinal co-pathology.

Patient data collected included age, gender, duration of symptoms, lens status, diagnosis (VMT only or VMT with the presence of a MH ≤ 400 µm), pre- and post-operative best-corrected visual acuity (BCVA) logMAR and MH minimum linear diameter (MLD) size.

**Fig. 1:**
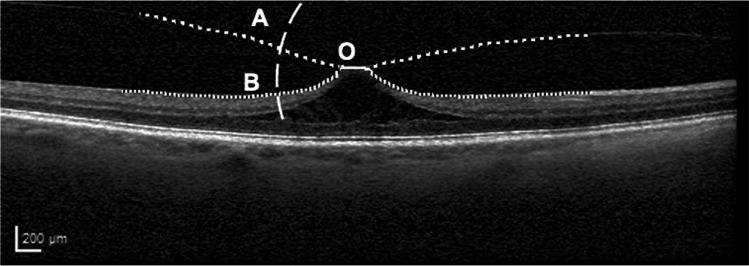
Example for the analysis of the length of vitreomacular adhesion (confluent line), and the angle of the nasal and temporal posterior vitreous insertion to the macula between the retinal (small-dotted lines) and vitreous (large-dotted lines) surfaces. The adhesion point (here left end of the confluent line) was used as the fixed point. A for the vitreous and B for the retinal surface were found between them and a circle centre at O. Then a straight line across O was fit by using least square method on the points along the choroidal surface between A and O; a regressed line could be found for the retina in the same way. Finally, the angle was calculated between the two regressed lines

Furthermore, the length of vitreomacular adhesion (VMA) and the immediate angle of the nasal and temporal posterior vitreous insertion to the macula in horizontal raster line OCT scans were analysed (Fig. 1). For each image, the VMA (confluent line) was annotated together with the retinal (small-dotted lines) and vitreous (large-dotted lines) surfaces. For the analysis of the detached vitreous angle (angle of vitreous insertion), the nasal and temporal adhesion points (ends on either side of the confluent line) were used as the fixed points. In order to estimate the angle between the retina and vitreous, first the intersection points, e.g. A for the vitreous and B for the retinal surface were found between them and a circle centre at O. Then a straight line across O was fit by using least square method on the points along the choroidal surface between A and O, a regressed line could be found for the retina in the same way. Finally, the angle was calculated between the two regressed lines. Figure 1 shows an example how A and B were found. The analysis program was written using Matlab R2019b (Mathworks, Natick, USA).

Main outcome measures were release of VMT and closure of MH confirmed by an OCT scan 4 weeks after PVL. Further outcomes were the formation of new MH, the progression of MH size of persistent MH, changes in BCVA to 4 weeks and 6 months after PVL (and PPV if required), the rate of secondary PPV required after PVL and retinal breaks or rhegmatogenous retinal detachment (RD).

All patients underwent intravitreal injection of 0.3 ml of 100% perfluoropropane (C_3_F_8_) with a 27-g or 30-g needle under topical or subconjunctival anaesthetic. An anterior chamber paracentesis was performed to control the intraocular pressure with or without adjunctive topical/oral anti-hypertensive medication. All surgeries were performed by trained vitreoretinal surgeons. Patients were instructed to posture face-down for 5 days following injection.

Variables were reported as mean (± standard deviation). For comparisons between continuous variables with normal distribution, Student’s *t*-test was used and Kruskal–Wallis and Fisher exact tests for non-parametric data. A generalised linear model was used with release of VMT and outcome after PVL (uncomplicated release of VMT, formation of a new MH, enlargement of a pre-existing MH) as dependent variables and pre-operative BCVA, age, symptom duration, lens status, angles of vitreous insertion and length of VMA as covariates and factors. The software used was SPSS 27.0 (SPSS, Inc., Chicago, IL).

Approval was obtained from the Ethic Committee at the Technical University of Munich. Under UK guidance, data collection is regarded as audit for the purposes of service evaluation and as such, ethical approval is not necessary. The study complied with the Declaration of Helsinki.

## Results

Forty-seven eyes of 47 patients were included (17 males and 30 females) with a mean age of 72 (± 8) (range 53–88) years. Mean symptom duration was 5.3 (± 5.4) months. At baseline, 33 patients had isolated VMT and 14 patients VMT with MH, with a mean MLD size of 139 µm (± 67) (range 47–289 µm). Thirty-seven patients were phakic and 10 patients were pseudophakic. Mean length of VMA was 700 (± 410) µm with a mean angle of vitreous insertion of 37 (± 15) ° on the nasal and 46 (± 19) ° on the temporal side.

### Release of VMT

Four weeks after PVL, overall VMT release rate was 35/47 (74.5%), with 24/33 (72.7%) eyes in the VMT only group and 11/14 (78.5%) in the VMT with MH group.

VMT was released in 25/37 (67.6%) phakic eyes and in 10/10 (100%) pseudophakic eyes (*p* = 0.03). There was no association of VMT release with age (*p* = 0.85), duration of symptoms (*p* = 0.71), presence of a MH (*p* = 0.65) or the length of VMA (*p* = 0.29). In addition, though there was a trend for larger angles of vitreomacular insertion to be associated with successful release of VMT, this did not reach statistical significance (nasal 47° vs 33°, *p* = 0.07, temporal 56° vs 41°, *p* = 0.09).

### Macular hole closure

In the VMT with MH group, 4/14 MH (28.6%) closed (all with release of VMT) 1 month after PVL. There was no significant difference in mean MLD size between MH that closed and those that did not (129 (± 109) vs.144 (± 50) µm, respectively) (*p* = 0.73).

### BCVA

Mean BCVA was 0.51 (± 0.25) logMAR prior to PVL and did not change significantly 4 weeks post-PVL (0.51 ± 0.33). At 6 months, however, BCVA improved by 0.14 (± 0.21) logMAR in patients with isolated VMT (*n* = 33) from 0.48 (± 0.24) at baseline to 0.34 (± 0.23) (*p* < 0.001). Similarly, in patients with VMT and MH (*n* = 14), BCVA improved by 0.16 (± 0.20) logMAR from 0.57 (± 0.27) to 0.41 (± 0.28) logMAR at 6 months (*p* = 0.008). Twenty-two of the 47 eyes, however, had undergone a PPV by then.

### Complications and indications for subsequent PPV

Twenty-two of 47 (46.8%) eyes required a subsequent PPV, 12/33 (36.4%) in the VMT only group and 10/14 (71.4%) in the VMT and MH group.

In the VMT only group, 9/33 (27.3%) patients failed to release VMT, of whom 3 declined further surgery. Reasons for subsequent PPV in the VMT only group were failure of VMT to release in 6 eyes, formation of a MH in 4/33 (12.1%) eyes with a mean MLD of 596 (± 44) µm (range 555–635 µm; two with and two without release of VMT) and 2 eyes for RD within 4 weeks after PVL.

In the VMT with MH group, 10/14 (71.4%) eyes underwent subsequent PPV for failure of the MH to close after PVL, and there was a significant progression in size from baseline 139 (± 67) µm to a mean MLD of 396 (± 130) µm (range 131–596 µm) at 4 weeks after PVL (236 (± 102) µm, *p* < 0.001). Two of the patients with failed MH closure also developed a RD. Enlargement of MH size was independent of lens status (*p* = 0.80), age (*p* = 0.53), duration of symptoms (*p* = 0.61) and length of VMA (*p* = 0.07). There was a trend for the temporal and nasal angles of vitreomacular insertion to be smaller in eyes that had enlargement of the MH following PVL compared to those that either did not develop a MH or in whom the MH did not enlarge though this did not reach statistical significance (*p* = 0.059).

Four of 47 (8.5%) eyes developed a retinal detachment within 4 weeks after PVL (2 VMT alone and 2 VMT with MH at baseline). The development of RD was independent of the lens status (*p* = 0.54), presence of a MH (0.09), age (*p* = 0.39), duration of symptoms (*p* = 0.40), length of VMA (*p* = 0.55) and nasal and temporal angles of vitreous insertion (*p* = 0.21 and *p* = 0.11, respectively).

Intraocular pressure spikes immediately after the injection were transient and medically controlled.

## Discussion

An alternative strategy for VMT release other than surgical PPV or ocriplasmin may have significant advantages if it avoids or mitigates the risks inherent in these treatments. The use of PVL for the treatment of VMT has been touted as one such potential modality, initially encouraged by high success rates in VMT release and also closure of MH ≤ 400 µm. [11–13].

Our VMT release rates (72.7% in the VMT only group and 78.5% in the VMT with MH group) are in line with previously reported high rates 4 weeks following PVL (40 to 100%) [14, 15, 18–20], and with a very recently published and to date largest prospective study, reporting release rates of 78% and 94%, respectively, at 24 weeks. [21] Of interest, in our study, there was a significantly higher VMT release rate in pseudophakic than in phakic eyes, which has not to our knowledge been identified before and is in contrast to ocriplasmin where pseudophakia is a recognised risk factor for failure of VMT release. This may be explained by the assumed mechanical rather than pharmacological mechanism that causes VMT release in PVL, potentially with the increased posterior chamber volume in pseudophakic eyes allowing greater tractional force on the vitreoretinal interface. However, only 10 of the 47 patients were pseudophakic and larger numbers may be needed to confirm this association. Furthermore, we identified a trend for the association of larger angles of vitreomacular insertion with successful release, which has already been reported by Paul et al [22] for the treatment with ociplasmin, and adds to the literature that there may be a particular anatomical configuration of VMT insertion which may help predict likely success of release for this procedure and may provide further insight for pharmacological VMT release; however, comparisons between mechanical and enzymatic vitreolysis have to be made with caution as the operating mechanism may differ significantly. One plausible explanation could be that smaller angles of vitreomacular insertion represent a more widespread adhesion of the posterior vitreous at the optic disc and the peri-foveal area, leading to failure of VMT release. The lack of association of VMT release with the length of VMA in our analysis may be explained by the fact the VMA length was rather short in all eyes with a mean of 700 µm and a rather narrow standard deviation of ± 410 µm.

The rate of MH closure following PVL varies greatly in previous studies (0–83%) and in our series is low (28.6%) despite the high rate of VMT release [14, 17, 18]. The largest literature review to date on PVL [20] reported a closure rate for stage 2 MH of 44% (20/45) at 1 month and 47% (21/45) at final follow-up. Following a single injection of a gas bubble, Mori et al. [17] demonstrated a VMT release rate of 95% (19/20) and a MH closure rate of 50% (10/20) at 1 month and Chan et al. [14] of 86% (43/50) and 53.3% (8/15), respectively. Mean MH sizes in previous studies are unknown. On the other hand, the results of the recent DRCR Retina Network Protocol AH demonstrated an equally low MH closure rate of only 29% as in our study, which is particular interesting since they had included only MHs ≤ 250 µm MLD size whereas we have included MHs ≤ 400 µm. [21] One potential explanation for the large discrepancy of the MH closure rates between various studies could be differences in the compliance with face-down posturing after PVL. Proper tamponade of the MH is crucial to allow MH closure; however, this is much harder to achieve with a small gas bubble as compared to a large bubble after PPV.

Of concern, we not only found a higher rate of MH formation after PVL (12.1%) in patients with isolated VMT than previously reported (0–6.67%) [14–16, 20] but also found the sizes of the newly formed MHs were all classified as large (range 555–635 µm) making subsequent surgical closure of the holes potentially challenging, though all four did surgically close subsequently. This raises into question the perception that PVL is a relatively “low-risk” strategy that can be undertaken as a preliminary procedure for VMT release and that if unsuccessful, can be easily resolved by surgical vitrectomy with minimal adverse consequence.

In addition, the current study also is the first to investigate the change in MH size following PVL demonstrating a significant enlargement of pre-existing MHs for those that failed to close after PVL. Ten of 14 eyes with MH at baseline progressed in size by a mean of 236 µm with 8 of those having transformed into a large MH > 400 µm, again emphasizing the potential visual cost of PVL as a preliminary treatment modality prior to definitive treatment with surgical vitrectomy.

VMT release rates reported after the treatment with ocriplasmin (26.5–47.4%) [5, 6, 10, 23–26] are lower than those after PVL with similar rates of non-surgical MH closure (30–58.3%) [10, 23–25], and similar rates of new MH formation (0.7–15.8%). [10, 23, 24].

Interestingly, none of the large trials on ocriplasmin has analysed the magnitude of MH size progression after failed treatment, with only the INJECT study [24] reporting an overall “potential” incidence of new (5.9%) or worsening (18.9%) MH, which lies well below the rates in our study (12.1% and 71.4%, respectively).

RD is a well-recognised complication of PVL for VMT release with many previous reports demonstrating a relatively low rate (0–2.2%) [14–16, 18–20]. However, in our study, RD occurred in 8.5% of the total cohort within a 4-week period, and 12% in the DRCR Retina Network Protocols developed a retinal detachment or tear within 24 weeks, which raises further significant concerns on the safety of PVL for VMT release and was the reason for the early termination of their study.

The rates of retinal tear or detachment formation after ocriplasmin (0.2 to 1.9%) [5, 6, 10, 24, 25, 27] are within the range of those previously reported after PVL but significantly below the rates we and the DRCR Retina Network study observed, potentially due to relatively small numbers of patients included in all available studies on PVL. Intuitively, if a technique has better overall success at VMT release, one might expect it to carry a higher risk of RD due to peripheral retinal traction.

Despite the high incidence of adverse events, the improvement of BCVA in our and Chan’s series on PVL was similar to those reported after ocriplasmin from baseline to final visit at 6–24 months (3.5–9.8 letters for the overall cohorts [10, 24, 25, 27], 3.2–7.7 letters for those with isolated VMT [10, 25] and 3.9–12.2 letters for those with a MH at baseline [10, 25]).

PPV remains far superior to both PVL and ocriplasmin in terms of release of VMT and closure of MH, due to the complete removal of the posterior vitreous, the ability to peel the internal limiting membrane and achieve a near 100% gas fill. Furthermore, improvement of BCVA after primary PPV for MH < 400 µm has been reported as high as 26 letters (from 0.75 (± 0.31) to 0.23 (± 0.18) logMAR) [28]. However, the burden of surgery may be significant particularly for elderly patients and a simpler, less invasive and more cost-effective strategy that confers good VMT release, even if less than the PPV reference standard is still a desirable goal, provided it does not compromise final visual outcome.

Our study has several limitations. It is retrospective and the numbers included are modest, and due to the high rate of MH formation and failure of MH to close a large proportion of patients underwent rescue PPV after 4 weeks, which prevented analysis of potentially further VMT release between 1 and 6 months after PVL.

Prospective large randomised controlled trials are needed to compare the outcomes, in particular also visual outcomes, of patients undergoing pneumatic or enzymatic vitreolysis with rescue PPV if needed versus primary PPV.

In summary, our study demonstrated that despite a high rate of VMT release following PVL, this technique was associated with a significant rate of complications with new large MH formation following VMT release, a significant enlargement of the majority of pre-existing MHs and a relatively high rate of RD following this procedure.

PVL has originally emerged as a low-invasive alternative to primary PPV. However, for most patients that developed above complications requiring consecutive PPV, PVL may actually have reduced their initial prognosis, which may have been better after primary PPV.

Based on the results of our study and the results of the DRCR Retina network study [21], PVL appears to carry considerable risk, which the treating surgeon needs to bear in mind when deciding the optimum treatment for these patients.
